# Microbial Phytases: Properties and Applications in the Food Industry

**DOI:** 10.1007/s00284-023-03471-1

**Published:** 2023-10-17

**Authors:** Hanane Joudaki, Negar Aria, Roya Moravej, Mohamadreza Rezaei Yazdi, Zarrindokht Emami-Karvani, Michael R. Hamblin

**Affiliations:** 1 Department of Microbiology, Falavarjan Branch, Islamic Azad University, Isfahan, Iran; 2 Department of Microbiology, School of Biology, Collect of Science, University of Tehran, Tehran, Iran; 3 Department of Biology, Sanandaj Branch, Islamic Azad University, Sanandaj, Iran; 4 Department of Microbiology, Faculty of Life Science, North Tehran Branch, Tehran, Iran; 5 Laser Research Centre, Faculty of Health Science, University of Johannesburg, Doornfontein, 2028 South Africa; 6 Radiation Biology Research Center, Iran University of Medical Sciences, Tehran, Iran

## Abstract

Microbial phytases are enzymes that break down phytic acid, an anti-nutritional compound found in plant-based foods. These enzymes which are derived from bacteria and fungi have diverse properties and can function under different pH and temperature conditions. Their ability to convert phytic acid into inositol and inorganic phosphate makes them valuable in food processing. The application of microbial phytases in the food industry has several advantages. Firstly, adding them to animal feedstuff improves phosphorus availability, leading to improved nutrient utilization and growth in animals. This also reduces environmental pollution by phosphorus from animal waste. Secondly, microbial phytases enhance mineral bioavailability and nutrient assimilation in plant-based food products, counteracting the negative effects of phytic acid on human health. They can also improve the taste and functional properties of food and release bioactive compounds that have beneficial health effects. To effectively use microbial phytases in the food industry, factors like enzyme production, purification, and immobilization techniques are important. Genetic engineering and protein engineering have enabled the development of phytases with improved properties such as enhanced stability, substrate specificity, and resistance to degradation. This review provides an overview of the properties and function of phytases, the microbial strains that produce them, and their industrial applications, focusing on new approaches.

## Introduction

**Fig. 1:**
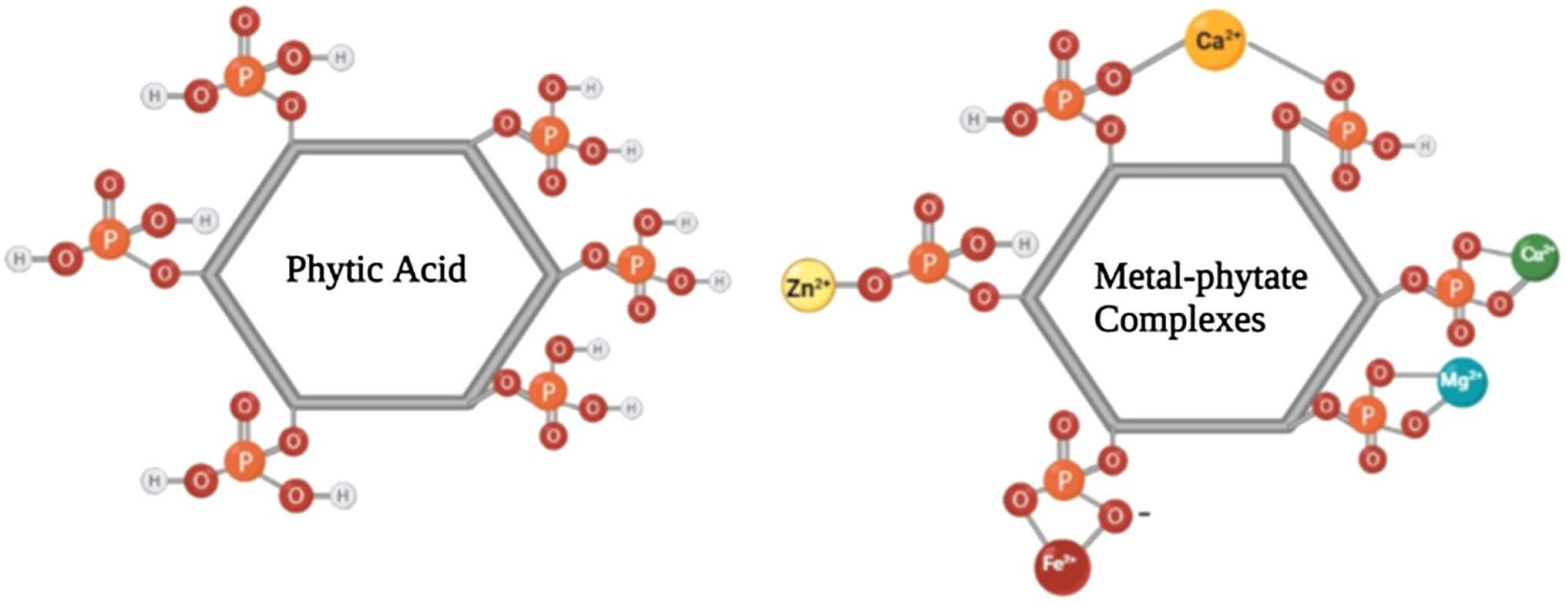
Phytate chelation of cations. Phytate is able to form stable chelates with Ca^2+^, Mg^2+^, Zn^2+^, Cu^2+^, Fe^3+^, and Fe^2+^ ions

Phytic acid or *myo*-inositol hexakis dihydrogen phosphate is mainly known as an anti-nutrient, which prevents absorption of other nutrients from the diet [1]. Phytic acid is the main store of phosphorus in plant foods. About 1–5% by weight of oilseeds, legumes, and grains is phytic acid, which affects the nutritional value of these foods. Phytic acid forms chelates with divalent or trivalent metal cations (Fe^+2^, Fe^+3^, Ca^+2^, Mg^+2^, Zn^+2^, Cu^+2^) (Fig. 1). Due to its pronounced negative charge, phytic acid forms complexes with proteins and enzymes, and disrupts their activity. The diet of domestic farm animals is based on plant sources, grains, and oilseeds, and therefore contains significant amounts of phytate that reduce the mineral uptake by monogastric animals [2]. On the other hand, the digestive system of these animals, like humans, lacks the enzyme phytase, and therefore the accumulated phytate prevents the uptake of metal ions from the animal diet [3]. Moreover, these non-absorbed minerals are released into the environment in large quantities through animal feces, leading to environmental pollution. Reducing the amount of phytic acid by chemical and physical methods affects other food constituents, and generally reduces the nutritional value of food products. The use of microbial phytase enzymes to reduce phytic acid in food can overcome these problems [4]. Phytase enzymes can hydrolyze phytic acid and phytates (phytic acid salts) and release mineral phosphate (Pi) and myo-inositol. Phytase enzymes degrade the anti-nutritional properties of phytate and prevent enzyme or protein complex formation with phytic acid, and the chelation of metal ions. Although some plant and animal sources of phytase enzymes have been reported, the main realistic sources of phytase are microorganisms [5]. Therefore, microbial phytases extracted from yeasts, fungi, or bacteria are principally used for commercial purposes [5]. Soil microorganisms have been widely screened as sources of phytase. However, new sources of phytase would be desirable for commercial use [6].

Dietary deficiencies in micronutrients, including zinc, iron, iodine, and selenium, are now a global health challenge for many populations around the world [7]. The addition of phytase to foodstuffs as a dietary supplement can break down existing phytate, without any adverse effects on other food components besides phytate. The methods used for pre-consumption food processing employing phytase can include soaking grains, boiling, fermentation, and flour processing, which are described in following sections [8]. Commercial phytase production and its use to decrease phytic acid in food is important to address global mineral shortages. The effects of phytase will depend on direct human bio-monitoring, food composition data, and knowledge of geographical variables impacting soil-to-crop transmission. According to current research, the phytase naturally present in mature cereal grains may digest phytate; however, the activity varies from species to species [9]. Furthermore, most of the phytases used as feedstuff supplements to enhance mature grains are histidine acid phosphatases (HAPs), which are microbial enzymes. Phytases are employed in dephytinization research and the production of myo-inositol phosphates, among other applications [10]. Phytases have been found in all three domains of bacteria, archaea, and eukaryotes. Nevertheless, due to their abundance in microbial sources, microbial phytases have been more often used in commercial applications, such as *Aspergillus niger* phytase, *Peniophora lycii* phytase, and *Escherichia coli* phytase [11]. Recent investigations have shown that in addition to bacteria and fungi being major sources of phytase, microalgae can also produce phytase, and can be used in the animal and human food industries [12]. Dietary nutrition, bread-making processes, animal feed supplements, transgenic crops, and probiotics isolation are some of the applications of phytases. The presence of phytases in soil reduces the likelihood of eutrophication [13]. The primary benefit of phytase supplementation of the soil is that it reduces the frequency with which chemical phosphate fertilizers are applied to the soil. Phytase can be used as an alternative to keep plants at the appropriate P level [13]. In addition to applications in the paper/pulp industries and soil remediation, semi-synthetic peroxidase systems are among other applications of phytase enzymes. This article will review the different types of phytases and their classification, their applications in the food industry, with an emphasis on new approaches, such as encapsulation, immobilization, genetic engineering, enzyme engineering, controlled fermentation, microbial consortia, and combinations with other enzymes [14].

## Phytase Classification

Phytases are classified according to the order in which the phosphate groups are released from phytic acid. These include the following: 3 phytases (EC 3.1.3.8), 4/6 phytases (EC 3.1.3.26), and 5 phytases (EC 3.1.3.72) [15]. Furthermore, there are three types of phytases classified as neutral, acidic, and alkaline. Acidic phytase is mainly found in a wide range of microorganisms and is most active at pH values between 2.0 and 6.0. Phytase extracted from *Aspergillus* species shows two different pH optimal values (pH 5.5 and 2.5). Neutral phytases have been reported in some bacteria and fungi [10]. In a study by 16 [16], a neutral phytase was extracted from *Aspergillus flavus*, which showed the highest activity at 45 °C and pH 7.0. Alkaline phytases were first detected in *Bacillus licheniformis*. Evidence suggests that these phytases are mainly dependent on Ca^2+^ for their activity. The active site conformation is governed by three low-affinity Ca-binding sites, while the other three high-affinity Ca-binding sites aid in substrate binding and structural stability [17]. The structure of alkaline phytase was revealed by X-ray analysis, as well as the amino acid sites where two phosphate groups and six Ca^2+^ ions may be bound. Ca^2+^ acts as a cofactor in this process [14]. The *Alcaligenes faecalis* bacterial phytase has a pH range of 7.0–8.0, which is similar to the pH of the gut environment of aquatic species; therefore, it might be utilized to increase the availability of P to fish, according to a study by Nassiri and Ariannejad [18]. Pollen grains from plants like *Lilium longixorum* and *Typha latifolia* contain an alkaline β-propeller phytase (BPP). *Bacillus amyloliquefaciens* and *Bacillus laevolacticus* are soil bacteria that also produce extracellular alkaline phytases. The optimal conditions for acidic phytase are pH = 4.5–6.0 and temperature 45–60 °C. The enzymes have an acidic pI, and a molecular weight approximately 40–70 kDa [19]. However, the range of pH values at which an alkaline phytate is active typically falls within the basic or alkaline pH range. Alkaline phytates are generally stable and more soluble at higher pH values. While the exact pH range can vary depending on the specific type of alkaline phytase and the experimental conditions, it is commonly observed within the pH range of 7–10 [20].

Phytases are also classified into cysteine phosphatase (CP), histidine acid phosphatase (HAPs), purple acid phosphatase (PAP), and β-propeller alkaline phytases (BPPs) based on their catalytic domains. Because all organisms require Pi, there are different kinds of catalytic mechanism which can decompose phytic acid into Pi [21]. For example, phytase activity has recently been recorded in two enzymes of the bacterial metallo-β-lactamase family [22]. HAPs are known as "fungal phytases." PAPs are found in plants, fungi and mammals, and BPPs in bacteria. BPPs are commonly found in *Bacillus spp*. [23].

## Sources of Phytases

Phytases are generated by bacteria, yeasts, and fungi and can be cell-bound or intracellular enzymes [24]. However, most fungal phytases are extracellular enzymes [24]. *Aspergillus* spp. are commonly used in the industrial mass production of phytases.

### Bacterial Phytases

The production of phytase has been reported in various bacteria, such as *Enterobacter* spp., *Bifidobacterium* spp., *Pseudomonas* spp., *Lactobacillus casei*, *Enterobacter* and *Serratia* [6], *Bacillus subtilis* subsp. *subtilis* JJBS250, *Escherichia coli*, and probiotic bacteria [25]. Submerged fermentation (SmF) is more often used in the commercial production of bacterial phytase than solid-state fermentation (SSF) [26]. There have been several efforts to isolate phytases from soil, plants, and animals. However, the production of phytase by bacteria is easier in terms of time and cost. Bacteria typically produce different extracellular phytases in different habitats [6]. One study isolated phytase from the bacterial strain *Serratia* sp. PSB-15. It had the capacity to dissolve phytate and was more thermostable than other bacterial phytases. Jain and Singh [27] isolated phytase from a soil sample of the phytase-producing bacterium *B. subtilis* subsp. s*ubtilis* JJBS250. *Bacillus nealsonii* ZJ0702 and *B. amyloliquefaciens* produce the most thermostable phytases that can withstand temperatures between 90 and 100 °C. BPPs with the ability to break down phytate are present in many rhizospheric bacteria. BPP was also detected in *P. fluorescens* JZ-DZ1 and has been proposed as a potential stimulant for phosphorus absorption in plants [28]. Bacterial phytases release a variety of compounds that increase access to sugars, Pi, amino acids, and soluble proteins, thereby improving soil nutritional conditions [27].

After microorganisms have been grown during submerged fermentation to produce phytase, they are typically used by the industry in one of the following forms. Firstly, in liquid fermentation broth, the broth contains the phytase-producing microorganisms, along with other metabolites, and can be used directly in certain applications. It may undergo further processing steps such as filtration or centrifugation to remove solid particles or impurities before use [29]. Secondly, in dried biomass, the microbial biomass obtained from the fermentation process is dried and transformed into a powdered or granulated form. This dried biomass retains the phytase activity and can be used as an additive to various food or feed formulations [29]. Thirdly, in concentrated enzyme extract, the fermentation broth can undergo downstream processing steps, such as filtration, ultrafiltration, or chromatography, to extract and concentrate the phytase enzyme. This concentrated enzyme extract, which may be in liquid or powder form, can then be used directly or further processed for specific applications [30]. Fourthly, encapsulated or immobilized enzymes, where the phytase enzyme is encapsulated or immobilized onto solid supports or carriers, such as beads, particles, or fibers. This allows for controlled release and prolonged activity, enabling its use in specific food-processing operations or as a feed additive [31].

The choice of formulation depends on the intended use, stability considerations, and the processing requirements of the industry. Each form offers advantages and may be chosen based on factors, such as ease of handling, storage stability, dosing flexibility, and compatibility with the intended food or feedstuff.

### Fungal and Yeast Phytases

Filamentous fungi like *Aspergillus, Mucor*, and *Penicillium* are responsible for the production of most microbial phytases. Fungi or yeasts that produce phytases include the following: *Sporotrichum thermophile*, *Penicillium purpurogenum*, *Aspergillus oryzae*, *Humicola nigrescens*, *Aspergillus flavus*, *Zygosaccharomyces*, *Pichia kudriavzevii*, and *S. cerevisiae* [32]. Fungal phytases can function at high temperatures and across a wide spectrum of pH values. *Sporotrichum thermophile* produces a phytase stable at pH 6.0 and 45 °C. *Thermophila myceliophthora* had phytase activity at 45 °C and 70% moisture. Phytase activity of *Thermomyces lanuginosus* TL-7 was also reported during growth on wheat bran [33].

Studies indicate that phytase production largely depends on the amount of Pi in the substrate used to induce phytase production. Gaind and Singh [16] reported the production of phytases by *Aspergillus flavus* using mustard cake for SSF growth. Fungal phytases are mostly extracellular. The growth of fermented yeasts in liquid and solid environments containing phytate as the sole source of P encourages phytase formation [34]. Also, mixed substrates (sugarcane bagasse and wheat bran) have been used to produce phytase in *Sporotrichum thermophile* [35]. SSF was also used in the production of phytase by *Aspergillus niger* 7A-1 isolated from triticale crop lesions [36]. Phytase production was reported under SmF conditions by *Saccharomyces cerevisiae* in a phytate medium using sucrose and aspartic acid as sources of carbon and nitrogen. At present, the most common commercial production of phytase is in fungi and yeasts, because they are an important source of extracellular phytase enzymes [37].

### Varieties of Microbial Phytase

Phytases function as recyclers of phytate and P in the environment. In general, according to the catalytic mechanisms and amino acid sequences, phytases can be divided into four categories: CP, HAP, PAP, and BPP. To determine the classification dependence among microbial phytases (HAP, CP, and BPP), a phylogenetic tree was designed using 16S rRNA genes [38]. Phytase-producing microorganisms have been identified in a variety of habitats. For instance, HAP-like sequences are found mainly in enteric and phytopathogenic microbes. There are more CP-like sequences in pathogenic, enteric, and free-living microbes. BPPs have been reported in different classes of microbes, including archea, bacteria, and eukarya [39]. A phytase has been reported in the halophilic archaea class of euryarchaeota, as an example of the most primitive microbes.

BPP activity has been reported in numerous bacterial phyla, including *Actinobacteria, Armatimonadetes*, *Bacteroidetes, Chlorobia, Cyanobacteria, Deinococcota, Firmicutes, Planctomycetes,* and in the eukaryotic phyla *Ascomycota* and *Basidiomycota*. The largest population of phylogenetic BPP bacteria occurs in the *Bacillus* and *Bacillus*-derived genera (BBDG) [40], while the lowest microbial population is found in the *Cyanobacterial* phylum. Examples of these bacteria are *Anabaena cylindrica* strain AFZ60317 and *Cyanothece* sp. strain WP_015957312. BPP activity has been observed in the bacterial phyla, *Firmicutes, Rubrobacteria, Nitriliruptoria, Coriobacteria, Actinobacteria, and Acidimicrobiia* [39]. Members of the phylum *Bacteroidetes* are relatively rare and often occur in extreme habitats. BPP activity in *Bacteroidetes* has been found in *Chlorobia, Deinococcota, Proteobacteria, Basidiomycota*, and *Ascomycota* [41]. Several niche-specific BPP microbes have also been reported, for example, *Bacillus aryabhattai*, *Bacillus amyloliquefaciens, Bacillus psychrotolerans*, *Pichia pastoris* isolated from thermal hot springs [40], *Pseudomonas* sp, *Pseudomonas nyackensis*, and *Rhodotorula mucilaginosa* isolated from cold habitats [42].

In addition to niche-specific microbes, BPP-producing microbes have been isolated from specific host plants, for example. *Serratia* sp. WRFC90 from saffron, *Rahnella* sp. JN27 and *Serratia* sp. WRFC90 from saffron, *Rahnella* sp. JN27 and *Pseudomonas putida* ZJF-G2 from rhizospheric soil of cereal crops, *Rahnella aquatilis* VRT-251 from *Eucalyptus*, *Paenibacillus* and *Bacillus* sp. from various pasture plants, *Klebsiella* sp. ASR1 from rice, *Enterobacter ludwigii* CL2 from wheat, and *Enterobacter cloacae* PYPB08 from leguminous crops [39]. Endophytic microbes producing BPP, such as *Bacillus tequilensis*, *Enterobacter ludwigii, E. cloacae, E. amnigenus*, and *Serratia grimesii* were reported to be present in soybeans, ginseng, and garlic. Bacterial strains producing BPP in aquatic environments have also been identified. For example, *Streptomyces, Prostachochloris, Glebobacter, Flavobac trim, Desulfuromonas*, and *Anabna* have been isolated from the gut of a fish (*Ctenopharyngodon idellus*) [43].

### Plant Sources of Phytase

The majority of plant phytases are HAPs, with an optimum pH of 4.5–6.0 and a temperature of 38–55 °C. Plant phytases belonging to the HAP family were formerly referred to as 6 phytases. Recent evidence suggests that some plant phytases (Lupin LP11 and LP12) start the orthophosphate hydrolysis at the D-3 location of the inositol ring [44]. Alkaline phosphatases, or PAPs, are present in certain other plants. The phytase from lilly pollen had an optimum pH of 8 and a temperature of 55 °C. This enzyme had a limited substrate specificity, with D-Ins (1, 2, 3) P3 as the end product, and was activated by calcium and inactivated by EDTA. Hegeman identified a phytase gene from sprouting soybeans that had little in common with HAPs, but a lot in common with PAPs, which had a binuclear Fe(III)–Me(II) core at the active site. The enzyme worked best at a pH of 4.5–5.0 and a temperature of 58 °C [45].

Phytase activity has been found in grains, legumes, and oil seeds, as well as dietary fruits and vegetables such as avocados and scallion leaves. Wheat, spelt, rye, barley, and triticale are examples of cereal grains with high phytase activity, which can be higher than 5000 units/kg. Animals have been fed with these grains and their by-products as a source of plant phytase [46]. To make effective use of the inherent phytase activity contained in plant foods, industrial or domestic processing methods such as germination, fermentation, and soaking can be used [47].

In addition to lupins, several other crops, cereals, and pseudo-cereals possess natural microbiota that can be utilized as a source of phytase. These native microbiota consist of diverse microorganisms, including bacteria, fungi, and yeasts, which naturally inhabit the rhizosphere (soil surrounding plant roots) or are present on the surface of these crops. Cereals such as wheat, barley, rice, maize, and oats have all been found to harbor-specific microbial strains capable of producing phytase enzymes [48]. These microorganisms can colonize the root surface or reside within the surrounding soil, forming a symbiotic relationship with the plants. They can release phytase enzymes into the rhizosphere, which helps in the degradation of phytic acid, thereby increasing the availability of phosphorus and other nutrients for the plant [25]. Pseudo-cereals, including quinoa, amaranth, and buckwheat, also have a similar association with native microbiota that possess phytase activity. These microbiota contribute to the breakdown of phytic acid in the soil or on the plant surfaces, aiding the release of phosphorus and improving nutrient absorption [49]. The presence of native microbiota with phytase activity in these crops and pseudo-cereals highlights their potential for enhancing nutrient availability and plant growth. Harnessing the beneficial effects of these native microbial communities can have significant implications for sustainable agriculture and the development of crop varieties with improved nutrient uptake efficiency [50]. Further research is being conducted to identify and characterize the specific microbial strains involved in phytase production within different crops and pseudo-cereals. Understanding the composition and function of the native microbiota associated with these plants will provide valuable insights into the manipulation and optimization of phytase activity, ultimately contributing to enhanced nutrient utilization and agricultural productivity.

### Phytase in Animal Tissues

Although phytase activity has been identified in some tissues from numerous animal species, none of the animal-derived phytases have been fully characterized at a molecular level [51]. Pigs, for example, are known to have high phytase activity within their digestive organs, particularly the stomach and pancreas. Several of these animal enzymes have an optimum pH in the neutral to alkaline range, and a Km for phytate ranging from 0.03 to 2.6 mM. Maenz (1998) discovered that phytase was present in the brush border vesicles of chicken intestine with a pH optimum of 5.5–6.0. Another study identified phytase in hybrid striped bass with an optimal pH of 3.5–4.5 [52]. Even though phytases have been isolated from the intestinal brush border membrane, the relatively inexpensive addition of exogenous phytase in feedstuffs may overshadow their practical use for increasing the availability of dietary phytate-phosphorus to simple-stomached animals [52]. The majority of phytase activity observed in the large intestine or rumen comes from commensal microbes. The presence of phytase within animal tissues suggests these enzymes have a role in aiding the digestion and utilization of phytate-bound phosphorus in animal diets. The phytase enzymes in these tissues can hydrolyze phytic acid, releasing phosphate and other nutrients that are otherwise unavailable for absorption [53]. This natural phytase activity helps enhance the digestion and utilization of dietary phosphorus, which is essential for animal growth and development. While the use of animal tissues as a commercial source of phytase is less common compared to microbial sources, their potential application has been explored to some extent. Researchers have investigated the extraction and purification of phytase enzymes from animal tissues for use in animal feed formulations [54]. This approach can provide a natural source of phytase that complements microbial phytases and offers additional benefits in specific animal production systems. It is important to note that the utilization of animal tissue-derived phytases requires careful consideration of factors such as availability, extraction methods, regulatory considerations, and potential allergenicity. Furthermore, the use of animal tissue-derived phytases in food production may have ethical and cultural implications that need to be addressed.

## Phytase Applications

Compounds derived from myo-inositol plays an important role in cellular signaling pathways. Phytases are used in industry for the production of myo-inositol, and can also be used to produce ethanol from maize. Phytases are also used to biodegrade organophosphate pesticides like monocrotophos, methyl parathion, and chlorpyrifos. By spraying *A. niger* NCIM 563 fungal phytase onto *Capsicum annum* L plants pretreated with chlorpyrifos, over 72% of the pesticide could be destroyed at pH 7.0 and 35 °C. Most of the applications of phytase in industrial research are related to animal nutrition and more than half of the food enzymes used for animals are phytases [22] (Table 1).

**Table 1:** Recent advances in microbial hosts for phytase production

Host	Gene source	Gene	References
*K. lactis GG799*	*A. niger*	phyA	[88]
*Chlamydomonas reinhardtii*	*E. coli*	appA	[89]
*Chlamydomonas reinhardtii*	*A. niger*	PhyA	[90]
*P. griseoroseum PG63*	*P. chrysogenum*	phyA	[91]
*L. lactis*	*E. coli*	appA	[92]
*Saccharomyces cerevisiae*	*A. niger*	phy A	[93]
*B. subtilis*	*B. subtilis*	phy	[94]
*P. pastoris*	*P. lycii*	phy	[95]
*P. pastoris*	*B. subtilis*	phyC	
*P. pastoris*	*E. coli*	appA	
*P. pastoris*	*S. thermophile*	rSt-Phy	
*P. pastoris*	*E. coli*	appA	
*P. pastoris*	*A. niger*	phyA	
*P. pastoris*	*C. amalonaticus*	phy	
*E. coli BL21 pLysS*	*P. agglomerans*	PaPhyC	[96]
*E. coli Bl21 (DE3)*	*Y. intermedia*	appA	
*B. subtilis 168*	*B. subtilis*	PHY	
*E. coli DH5a*	*E. sakazakii 4194.4532*		
*P. pastoris*	*P. anomala*	Pphy	[97]
*P. pastoris*	*E. coli*	appA 1.2	
*P. pastoris*	*Lilium longiflorum*	LlALP2	
*P. pastoris*	*C. amalonaticus*	phy 2119	

### Industrial Applications of Phytase

Various myo-inositol phosphates play an important role in cellular signaling pathways. Phytases are used in industrial processes for the production of myo-inositol phosphates, and are also used to produce ethanol from maize [55]. Phytases are also used to enhance the biodegradation of organophosphate pesticides such as monocrotophos, methyl parathion, and chlorpyrifos. When the fungal phytase from *A. niger* NCIM 563 was spayed on *Capsicum annum* L pretreated with chlorpyrifos, over 72% of the pesticide was destroyed at pH 7.0 and 35 °C. Many of the applications of phytase in research are related to animal nutrition, and more than half of the food enzymes employed in animal feedstuffs are phytases [22].

### Application of Phytase in Food Industry

Monogastric animals have only low phytase activity in their gastrointestinal tract. Food for birds and pigs is often based on maize, sorghum, and wheat, all of which are rich in phytic acid. Therefore, because of the poor availability of Pi, inorganic cations, and organic molecules, the growth of these animals may be seriously threatened [56]. Thus, feedstuffs for pigs and poultry should be supplemented by adding Pi [57]. To use phytases in the food industry, they need to be stable and be able to function at gastric pH and should survive the digestive process to some extent. Phytases must have thermal stability because the food production process often occurs at a temperature of approximately 65–90 °C. However, to prevent the inactivation of phytases, the enzyme may be added to the food following the heat treatment processing. Microbial phytases are also sometimes used in fish feeds [58].

Because wild microbial strains sometimes produce low amounts of proteins, genetically engineered strains are commonly used for phytase production. One commercial product produced by BASF is called Natuphos® E, a phytase derived from *A. niger* var. *ficuum*. Natuphos is a 3-phytase and is mainly used in the field of pig nutrition. Natuphos E was designed to be resistant to pepsin, pH, and temperature [22]. Other industrial phytases include Axtra Phy (made by DuPont) and Ronozyme (made by DSM/Novozyme). Axtra Phy is prepared from *Buttiauxella* sp and has a higher relative activity at pH 5.0, 95 °C, and is resistant to pepsin [22]. Ronozyme Hiphos and Ronozyme NP are also commercial products based on phytase enzymes from *Citrobacter braakii* and *Peniophora lycii*, respectively. In general, the base quantity discharged is proportional to the enzyme concentration used [59]. A comparison between Ronozyme NP and Natuphos phytases revealed that Ronozyme NP had a higher Kcat value for phytate at pH 3.0–5.0 and 37 °C, but had a greater sensitivity to proteases.

**Fig. 2:**
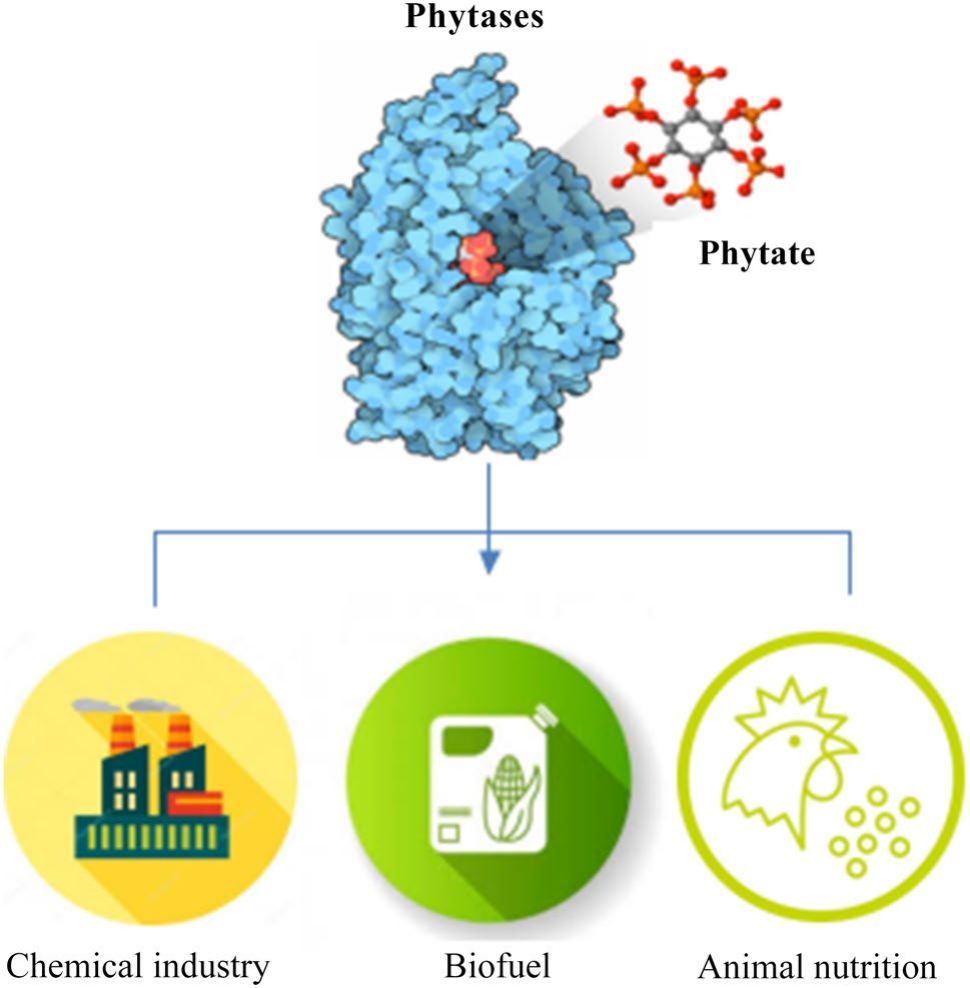
Phytases for industrial applications. Phytase enzymes and phytate have a variety of applications in the chemical, biofuel, and animal nutrition industries

Generally, feed enrichment with 250–500 FTUs (phytase units) per kilogram is recommended; however, the use of more than 500 FTUs could improve the benefits of adding phytase. Many studies have demonstrated that a high calcium diet influences phytase activity. This is because calcium interacts with phytate molecules and makes them less accessible to phytase cleavage [60]. Given the widespread use of fungal phytases in industrial environments, more studies are needed to identify efficient genes and proteins coding for phytase enzymes in filamentous fungi (Fig. 2).

### Application of Phytase and Phytate in Human Nutrition

Plant food sources are the main source of human food worldwide, providing carbohydrates, vitamins, dietary fiber, and proteins. In addition, these food sources also contain non-nutritious materials, such as phytic acid, which could be a concern in human health and diet management [61]. As mentioned above, phytate forms chelates with divalent cations to produce an insoluble material. Therefore, phytate has a negative effect on the absorption and digestion of these minerals (iron, calcium, zinc, and magnesium) by both humans and animals [62]. Phytates or salts of phytic acid exist as salts of divalent cations and accumulate in plant seeds as the main form of storage of phosphate and inositol. The lack of phytase in the animal intestine leads to humans, birds, and animals being unable to absorb phosphorus and minerals from their diet. Because phytate is a strongly negatively charged compound that exists across a wide pH range, its presence in the diet reduces the bioavailability of divalent and trivalent mineral ions [63]. On the other hand, the formation of phytate protein complexes reduces the enzymatic activity, solubility, and proteolytic susceptibility of proteins. Therefore, phytate is known as an anti-nutrient compound.

The phytase enzyme is able to hydrolyze phytate, and its addition is a solution widely considered in the food and animal feedstuff industry. Phytate consumption, despite its detrimental impacts on human health, has been reported to have some positive effects. The investigation of communities eating vegetarian-type diets has revealed a reduced cancer incidence, suggesting that phytate may have an anticarcinogenic effect [63]. The metal binding property of phytate means that it can also have an anti-oxidant function, limiting the production of hydroxyl radicals by Fenton type reactions, thus helping cells maintain homeostasis, and can be considered as a natural dietary anti-oxidant [64]. Dietary phytate may have health advantages for diabetics because it decreases blood glucose levels by delaying stomach emptying and decreasing the rate of starch breakdown. Phytate has also been found to influence insulin secretion [65]. Phytate is thought to be able to protect against heart disease by lowering cholesterol and triglycerides and inhibiting blood clotting. It may also help to avoid the formation of kidney stones [66]. Phytate is sometimes used as a complexing agent to remove heavy metal ions from the body in cases of poisoning. In vitro experiments have shown that incubating phytic acid with HIV-1 infected T cells, reduced the multiplication of HIV-1 virus [67].

The phytate-hydrolyzing enzymes have a variety of uses within the food industry. Mineral absorption in the small intestine is adversely affected by phytate. Phytase has the potential to produce bread with a reduced phytin content. Phytase enhances bread quality in two ways: firstly, it increases the nutritional value by lowering the phytate level [68], and secondly, it stimulates endogenous α-amylase activation by increasing calcium availability. Similarly, when wheat bread rolls contained fungal phytase, non-heme iron absorption in humans was doubled compared to bread that had not been treated with phytase [69]. Because soymilk contains a significant amount of phytate (0.56%), phytase can be added to prepare phytate-free soyabean milk [70]. Porridge was made from wheat, rice, oats, maize, sorghum, or wheat-soy flour blends that were evaluated for iron absorption in humans. When porridges were made with water, iron absorption improved, but the use of milk had no effect [71]. According to reports, microbial phytase might speed up the steeping process employed in maize wet milling, therefore enhancing the production of corn steep liquor [72]. Tarhana, a traditional Turkish fermented and dried grain meal, is high in minerals with good bioavailability (Ca, Mg, and K). In addition to using baker's yeast as a phytase source, fermentation under increasing acidic conditions resulted in a considerable drop in phytic acid content and an increase in overall quantities of minerals and proteins. Chapathi (also known as roti) is a popular snack in regions of India. Chapathis are made using whole-wheat flour, which contains a high level of phytic acid. Phytate levels can be reduced by using a mutant strain of the yeast *Candida versatilis* as a source of phytase during the production of chapathi dough, which decreases phytate levels by 10–45% [73].

Human-safe probiotic microorganisms such as *Lactobacillus*, whose consumption has long been known to be healthy for livestock and humans, can be used to reduce phytate levels in human food. Nevertheless, the phytase activity of different probiotics has not been fully investigated yet. Recombinant phytase production by gene transfer into these strains is another method being studied for the production of phytase for the human food industry [74].

### Phytase in the Production of Myo-Inositol Phosphates

Mobilization of Ca from intracellular reserves as well as transmembrane signaling transduction is carried out by phospholipids and various types of inositol phosphates. For the synthesis of different inositol phosphate structures, phytase is essential. Myo-inositol phosphates can act as enzyme stabilizers, enzyme substrates, and enzyme inhibitors and may even have therapeutic uses in future. For their physiological activity, the number and location of the phosphate groups on the myo-inositol ring are extremely important [75].

### Phytase and Plant Growth

In many parts of the world, because of a growing deficiency in soil phosphorous, plant growth and crop productivity are decreasing. Because phosphorus produces insoluble compounds by reacting with compounds in soil, more than 80% of the organic P in the soil is made up of plant debris and compost materials. Pi is present in apatite combined with Ca, Fe, and aluminum phosphate (Al) and is also adsorbed on clay particles. Although total phosphorus could be present in a sufficient amount of 0.05%, only 0.1% of this 0.05% may be absorbed by the plant as orthophosphates [76]. Chemical phosphate fertilizers are widely used as a primary source of P for increasing crop productivity. However, most phosphorus-based fertilizers rapidly become insoluble after application, causing damage to the environment. Furthermore, the low quality of phosphate, the high cost of transport, and the high price encourage the replacement of chemical fertilizers with cheap and more cost-effective compounds. One solution to the phosphorous deficiency problem is to dissolve mineral and insoluble phosphates using psychrotolerant phosphate solubilizing bacteria (PSB). Special consideration has recently been given to the use of PSBs as a type of biofertilizer. In addition to being eco-friendly and profitable, PSB also have beneficial effects on plant growth and yield as well as soil fertility. PSBs release organic acids which dissolve phosphorus and provide it to the plants. An alternative strategy is to use phytases to release phosphorus from phytates and make it accessible to plants [4].

Recent studies have demonstrated that if phytase or a phytase-producing microbial strain is added to the soil, more phosphorous is available for the plants. This is an effective and sustainable strategy for improving the natural phosphorus resources in the soil ecosystem [77]. For example, *Bacillus subtilis* KPS-11, in addition to increasing soil organic phosphorus, also increased the growth of *Solanum tuberosum* L. The plant growth-promoting rhizobacteria (PGPR) *B. amyloliquefaciens* FZB45 secretes an extracellular phytase that promotes the growth of corn plants in phosphate-deficient conditions. The reason for the better growth is the breakdown of phytate by phytase activity leading to an increase in phosphorus and minerals vital to the plant [78]. Furthermore, the bacteria secrete a highly active phytase that acts as a biofertilizer to promote the growth of tobacco plants. These results indicate that the phytases secreted by microbial strains have a strong capacity to hydrolyze soil phytate, which increases phosphorus and therefore its absorption by plants. In summary, studies show that the absence or lack of sufficient active phytase in the vicinity of plant roots inhibits the uptake of phytate-P in the soil. Phytase-producing microorganisms may also be used to provide phosphorus for plant absorption and to increase productivity in the horticulture and agriculture industries [79].

## Future Outlook

In general, some phytase enzymes have recently been produced by recombinant methods in various microorganisms to increase the use of phosphorus present as IP6 in animal feedstuff. Expression systems for these recombinant enzymes can be employed in either prokaryotic or eukaryotic hosts. Although prokaryotic expression systems can be easily genetically engineered and require simple culture conditions, post-translational changes do not occur in these recombinant proteins. However, recombinant proteins produced in eukaryotic systems such as yeasts, plants, and fungal hosts do undergo these post-translational changes. Recombinant phytases, in addition to being beneficial for the environment and animal nutrition, could in future be used as anti-cancer agents in the human diet. IP6 is known to be a cancer chemical that is dephosphorylated at IP1-5 after reaching malignant cells. Because IP6 has protective effects in less phosphorylated forms, the discovery of new phytases could be useful for cancer research [80].

In recent years, new methods have emerged for the application of microbial phytases in the food industries, further enhancing their effectiveness and expanding their utility. These methods involve innovative approaches to optimize phytase activity, improve phytic acid degradation, and enhance the overall suitability of food products [81]. The following are some notable methods that have gained attention in this field. (a) Immobilization techniques involve the immobilization of microbial phytases onto solid supports or carriers to improve their stability, reusability, and ease of application. Immobilization techniques such as adsorption, entrapment, covalent binding, and encapsulation have been explored. Immobilized phytases exhibit enhanced resistance to pH and temperature variations, making them suitable for a wide range of food-processing conditions [82]. (b) Genetic engineering advances have allowed for the development of genetically modified microorganisms (GMOs) capable of producing phytases with improved properties. Using genetic manipulation, phytases can be engineered to possess enhanced thermal stability, pH tolerance, substrate specificity, and resistance to proteolytic degradation. These engineered phytases offer improved efficiency and reliability in food applications [83]. (c) Co-culturing and fermentation strategies using different microbial strains or employing mixed cultures in fermentation processes have been investigated to enhance the phytase production and activity. Synergistic interactions between microorganisms can lead to improved enzyme yields, increased enzyme stability, and enhanced phytic acid degradation. Co-culturing also allows for the production of multiple enzymes simultaneously, offering a more comprehensive approach to address various anti-nutritional factors in food [84]. (d) Nanotechnology-based approaches have been employed to enhance the delivery and effectiveness of microbial phytases in food matrices. Encapsulation of phytases in nanocarriers or nanogels improves their stability, protects them from environmental factors, and enables controlled release in the digestive system. Nanostructured materials can also be used to improve the bioavailability of phytase-treated food products by enhancing nutrient absorption in the gastrointestinal tract [85]. (e) Enzyme Cocktail Strategies combine microbial phytases with other enzymes or additives in enzyme cocktails for their synergistic effects on phytic acid degradation and nutritional improvement. The inclusion of complementary enzymes, such as proteases, carbohydrases, or other phytate-degrading enzymes, can lead to more efficient phytic acid hydrolysis and release of bioactive compounds. Enzyme cocktails offer a comprehensive solution to improve the nutritional profile and functional properties of food products [86]. (f) Process optimization techniques, such as ultrasound-assisted treatment, microwave-assisted processing, and high-pressure processing, have been investigated to improve the efficiency and speed of phytic acid degradation. These technologies can allow better enzyme–substrate interactions, accelerate enzymatic reactions, and improve the overall effectiveness of phytase treatment in food processing [87].

These new approaches for the application of microbial phytases in the food industry offer exciting opportunities to optimize enzyme performance, improve food quality, and enhance nutritional value. Continued research and development in these areas will further advance the field, leading to the development of novel and effective strategies for addressing the challenges posed by phytic acid and anti-nutritional factors in food.

## Conclusion

This study has summarized the current information in the area of phytases used in the food industry. The main use of phytases is as a feed supplement for livestock. However, this use has developed into the production of ethanol and the degradation of organophosphate compounds. Further research on other sources of phytase enzymes is of particular interest in extending their applicability in new industrial sectors. Moreover, research on the interactions between phytase and food is a lucrative area for further discoveries. The high demand for phytases opens new horizons for the discovery of catalysts with enhanced properties for industrial use.

## Data Availability

Data are available from corresponding author on reasonable request.
